# AGA induces sub-G1 cell cycle arrest and apoptosis in human colon cancer cells through p53-independent/p53-dependent pathway

**DOI:** 10.1186/s12885-022-10466-x

**Published:** 2023-01-02

**Authors:** Bou-Yue Peng, Abhinay Kumar Singh, Chun-Hao Chan, Yue-Hua Deng, Pin-Ying Li, Chun-Wei Su, Chia-Yu Wu, Win-Ping Deng

**Affiliations:** 1 Department of Dentistry, Taipei Medical University Hospital, 110301 Taipei, Taiwan; 2 School of Dentistry, College of Oral Medicine, Taipei Medical University, 110301 Taipei, Taiwan; 3 Stem Cell Research Center, College of Oral Medicine, Taipei Medical University, 110301 Taipei, Taiwan; 4 Division of Oral and Maxillofacial Surgery, Department of Dentistry, Taipei Medical University Hospital, 110301 Taipei, Taiwan; 5 Graduate Institute of Biomedical and Pharmaceutical Science, Fu Jen Catholic University, 242062 Taipei, Taiwan; 6 Department of Life Science, Tunghai University, 407224 Taichung, Taiwan

**Keywords:** p53-dependent/independent, AGA, CDK2, CDK6, Apoptosis, Cell cycle

## Abstract

**Background:**

Despite the advancement in chemotherapeutic drugs for colon cancer treatment, it is still a life-threatening disease worldwide due to drug resistance. Therefore, an urgently needed to develop novel drugs for colon cancer therapies. AGA is a combination of traditional Chinese medicine Antler’s extract (A), *Ganoderma lucidum* (G), and *Antrodia camphorata* (A); it contains a lot of biomolecules like polysaccharides, fatty acids, and triterpenoids that are known to exerting anti-oxidative, anti-inflammatory, anti-microbial and anti-tumor activities in oral cancer. In this study, we investigate AGA anti-proliferative, anti-metastatic and apoptotic activity to explore its anti-cancer activity against colon cancer cells and its underlying mechanism.

**Method:**

Here, *in-vitro* studies were performed to determine the antiproliferative activity of AGA through MTT and colony formation assays. Wound healing and transwell migration assay were used to evaluate the metastasis. Flow cytometry and protein expression were used to investigate the involved molecular mechanism by evaluating the cell cycle and apoptosis. The *in-vivo* anti-cancerous activity of AGA was assessed by xenograft mice model of colon cancer cells.

**Results:**

We found that AGA significantly inhibited the proliferative capacity and metastasis of colon cancer cells *in-vitro*. In addition, AGA induced cell cycle arrest in the sub-G1 phase through upregulating p21 and downregulating CDK2, CDK6 in SW620, and CDK4 in SW480 and HT29, respectively. Annexin-v assay indicated that colon cancer cells had entered early and late apoptosis after treatment with AGA. Furthermore, a mechanistic protein expressions study revealed that AGA in p53-dependent and independent regulated the apoptosis of colon cancer by downregulating the p53 protein expression in SW620 and SW480 cells but upregulating in a dose-dependent manner in HT29 cells and increasing the expression of Bax and caspase-9 to inhibit the colon cancer cells. In vivo study, we found that AGA significantly reduced the xenograft tumor growth in NOD/SCID mice with no adverse effect on the kidney and liver.

**Conclusion:**

Collectively, AGA has the potential to inhibit colon cancer through inhibiting proliferation, migration, and cell cycle kinase by upregulating p21 protein expression and promoting the apoptotic protein in a p53-dependent and independent manner.

**Supplementary Information:**

The online version contains supplementary material available at 10.1186/s12885-022-10466-x.

## Background

Colon cancer is the world’s third most commonly diagnosed cancer; it is the second most common cancer in females and third in males, respectively [1–3]. Cancer stages at the time of diagnosis negatively correlate with the patient’s survival since cell deregulation is often enhanced in the advanced stage of the disease, which limits the therapeutic strategies in surgery and radiation therapy alone [4, 5]. Deregulation of the cell cycle underlies the abnormal cell proliferation, migration, and apoptosis that characterize cancer. Cell cycle control losses make it a potential target for chemotherapeutic agents. 5-FU (5-Fluorouracil), xeloda (capecitabine), camptosar (irinotecan), eloxatin (oxaliplatin), and Lonsurf (trifluridine/tipiracil) are chemotherapeutic agents which commonly used in the treatment of colon cancer cell by arresting the cell cycle in S-phase [6]. Various studies reported that combinations of different chemotherapeutic agents could potentially inhibit cancer in terms of overall survival [7]. Emerging evidence does not support stronger efficiency in combined therapy of FU + oxaliplatin, FU + oxaliplatin + irinotecan, and capecitabine + oxaliplatin therapeutic agents, applied in treatment because of its potential to increase resistance and toxicity [8]. However, several studies reported that using chemotherapeutic agents in colon cancer patients has increased the overall survival by up to 20 months, resulting in chemotherapy being a potential backbone of colon cancer treatment [9]. In addition, cancer resistance to treatment with traditional chemotherapeutic agents is the main cause of poor efficacy or treatment failure. Therefore, there is substantial demand to search for novel combinations of different drugs to overcome these obstacles, which are safer to use and provide optimum therapeutic effects in colon cancer therapy.

The diversified lifestyle and western diet potentially concern the clinical community regarding colon cancer [10]. Moreover, the commonly associated high mortality and therapeutic resistance have exposed the fragility of its treatment. Considerable advances in the pathophysiological understanding of colon cancer have found a way to target therapies for its treatment. In particular, modulation of p53 pathways may interact with various negative regulators responsible for pathological events in acute and chronic colon cancer [11]. Mostly, p53 expression decrease and impairs its transcriptional functions, but sometime upregulated p53 expression may induce the oncogenic activities, contributing to apoptosis inhibition, chemoresistance, and creating a worse outcome. p53 in cancer therapy mainly causes the cell cycle arrest at the G1 phase by the induction of cyclin-dependent inhibitor, p21 [12]. Although downregulation of p53 protein expression is associated with decreased p21 expression, also many studies showed p53-independent induction of p21 [13]. Further, p21 regulates the cell cycle, inhibits cell proliferation and migration, and induces apoptosis [13]. Recent studies reported that p21 inhibits the CDK by non-catalytic inhibition of CDK2 from CDK4 complexes, and it also inhibits the CDK6 [14]. As we know, widely accepted that restoration of p53 function is a well-studied strategy in anti-cancer therapy, but even p53-based therapy is still not clinically available for cancer therapy. Moreover, various p53 target drugs are under clinical trial PRIMA-1^MET^ (APR-246; NCT03745716; Phase III) has a p53-independent inhibitory activity in the colon cancer cell, and the mechanism of antitumor activity of the mutp53 reactivator COTI-2 (NCT02433626; Phase I) is still not comprehensively understood in many cancers, including colon cancer [15]. Therefore, we also need to find a novel combination of a drug candidate that could target colon cancer by the p53-dependent or independent pathway.

AGA, is a combination of three traditional Chinese medicine that is Antler’s (A), *Ganoderma lucidum* (G), and *Antrodia camphorata* (A); it was first reported to have a potential role in the inhibition of oral cancer growth and progression [16]. Most researchers have found that Antler’s extract has therapeutic properties to improve the immune system and physical strength. Previous studies have shown that Antler’s exhibits an anti-tumor effect in glioblastoma cell lines while being also reveal a non-toxic effect in non-cancerous cell lines [17]. The most biologically active compounds in antler’s extract regulate the cell growth required for anther’s regeneration and thereby could reduce cancer in human or mice models [18]. In addition, *Ganoderma lucidum* is listed as herbal medicine in Chinese pharmacopeia and American pharmacopeia due to its anti-inflammatory, anti-diabetic, and immune-modulatory effects. It contains polysaccharides that show an anti-cancer effect by inhibiting cancer cell migration. It also reported that water extract of *Ganoderma lucidum* inhibits the cell motility in breast and prostate cancer in a dose-dependent manner [19, 20]. *Antrodia camphorata* is a fungus that uniquely grows in Taiwan, and is traditionally used as medicine for various diseases. Moreover, a study reported that ethanolic extract of antrodia has the ability to induce apoptosis and retards migration of hepatocellular carcinoma [21]. These traditional medicines separately have reported anti-cancer properties in different cancers, but their combined regimen has not yet been reported for colon cancer. Therefore, in this study, we investigated the *in-vitro* and *in-vivo* anti-tumor activity of the AGA cocktail of three traditional medicine and its underlying mechanism of p53-dependent/independent pathway against colon cancer.

## Material and method

### Cell lines and reagents

In our experiments, we used three different colon cancer cell lines HT29, SW620, and SW480, which were purchased from American Type Culture Collection (ATCC). SW620 and SW480 were cultured in RPMI medium (Gibco/Life Technologies, Waltham, MA, USA), and HT29 cells were in DMEM medium (Gibco/Life Technologies) with 10% fetal bovine serum (FBS; Corning; Merck, Kenilworth, NJ, USA, KGaA) and 1% penicillin/streptomycin (Sigma-Aldrich; Merck KGaA) and maintained in a 37 °C incubator with 5% CO2.

### Preparation of the Herbal Cocktail AGA

AGA extract was provided by Well Shine Biotechnology Development Co., Ltd., Taipei, Taiwan. It was a cocktail of Traditional Chinese medicines Antler’s extract (A), *Ganoderma lucidum* (G), and *Antrodia Camphorata* (A), it also contains a small amount of L-arginine and maltodextrin.

### Cell viability assay

Colon cancer cells SW620, SW480, and HT29 of 1000 cells per well were seeded in 96-well plates at 37 °C for 24 h. After that, the cells were treated with AGA extract (0, 10, and 20 mg/mL) and incubated for 48 and 72 h. Then, cell viability was determined by adding 25 µL MTT reagent (Sigma-Aldrich, St. Louis, CA, USA, Cat No. M5655) to each well and incubating for 4 h, followed by the addition of 150 µL of N, N-dimethylsulfoxide (DMSO) (Sigma-Aldrich, Cat. No. D5879). The plates were placed on a shaker to allow complete lysis of the cells and were read at 595 nm the following day [16].

### Colony formation assay

To evaluate the effect of AGA extract on colon cancer cells, the 500 cells of SW620, SW480, and HT29 were seeded in10 mm cell culture dishes and incubated at 37 °C. After 24 h of cell seeding, they were incubated with AGA extract (0, 10, and 20 mg/mL) and incubated for 14 days. The culture medium was changed after every 3 days with a fresh medium. Then, cells were washed twice with PBS, fixed with formalin for 15 min at 4 °C, and stained with crystal violet dye (0.1% *w/v*) at room temperature for 1 h. After staining, washed with distilled water, we counted the colonies with a colony counter machine (Suntex, Colony counter 570).

### Wound-Healing Assay

Six well plates were used in the wound healing assay. Briefly, colon cancer cells SW620, SW480, and HT29 were seeded (1.5 × 10^5^ cells/well) in six-well plates and incubated at 37 °C for 24 h. When cells reached 100% confluence, a straight line with the same width was scratched across the monolayer, and PBS was used for washing to remove non-adherent cells. The AGA extract concentration of 0, 10, and 20 mg/mL were used to treat these cells and incubated for 48 and 72 h. Three randomly chosen fields at the lesion border were used to calculate the percentage of wound closure.

### Transwell Migration and Invasion assays

*In-vitro* cell migration was examined using the 8 μm BD Falcon cell culture insert (BD Biosciences, NJ). The cells SW620, SW480, and HT29 were seeded (1 × 10^5^ cells/well) in their respective serum-free medium into the upper compartment whereas the lower compartment was filled with a respective medium containing 10% FBS. After 24, 48, and 72 h of incubation with AGA extract at 37 °C in 5% CO2. Cells on the lower compartment were stained with 0.1% crystal violet, and migrating cells were counted. Thereafter, through MTT assay, we determined whether the effects of AGA extract (0, 10, and 20 mg/mL) on cell migration were due to the inhibition of cell viability.

### Cell-cycle analysis

Effects of AGA extract (0, 10, and 20 mg/ml) were evaluated on the cell cycle of colon cancer cells (SW620, SW480, and HT29) after incubation for 48 h, cells were harvested to make a cell suspension and added 500 µL of propidium iodide (0.02 mg/mL, Sigma-Aldrich, 11,348,639,001) and 500 µL of RNase A (0.2 mg/mL, Sigma-Aldrich, 10,109,142,001), and incubated for 30 min in the dark. The samples were analyzed through flow cytometry, data acquisition and analysis were done using Sony SA3800 software, and G1, S, and G2/M (mitosis) phases percentages were determined in each colon cancer cell.

### Apoptosis assay

The apoptosis of colon cancer cells (SW620, SW480, and HT29) after being treated with AGA extract was determined by a PE Annexin V Apoptosis Detection Kit with 7-AAD (BioLegend, San Diego, CA, USA). Briefly, colon cancer cells were treated with AGA extract (0, 10, and 20 mg/mL) for 48 h. After treatment, cells were harvested and stained with PE Annexin V/7-AAD for 15 min, and analyzed by using a Sony SA3800 flow cytometer.

### Western blot analysis

Protein extraction and immunoblotting were performed as previously described [23]. Colon cancer cells were treated with AGA extract for 48 h, then protein was extracted from each cell after AGA extract treatment by using RIPA lysis buffer. The protein concentration of each cell extract was determined by the BCA method. Approximately 30 µg total protein were mixed with 6 x loading dye buffer and heated at 100^o^C for 10 min, followed by the separation in 10% SDS-PAGE gel, blotting on PVDF membrane, subsequently, the membrane was cut and incubated with primary antibodies p21 (BF683) mouse mAb, CDK2 (D5C10) rabbit mAb, CDK4 (D9G3E) rabbit mAb, CDK6 (92G2) rabbit mAb, p53 (D21H3) rabbit mAb, Caspase-9 (D10A8) rabbit mAb and β-actin (Millipore #MAB1501, 1:10000) for overnight at 4^o^C after blocking with 3% BSA, followed by incubation with secondary antibody (1:10000). ImageJ software was used to quantify the gray values of primary antibodies.

### Animal studies

All animal experiments were performed according to the Institutional Animal Care and Use Committee (IACUC) of Taipei Medical University (Approval no. LAC-2019-0307). A 6-week immunodeficiency (NOD/SCID) mice were purchased from BioLAS Co., Taipei, Taiwan, and the animals were kept under pathogen-free conditions and fed with food and water. To examine the tumorigenicity, 2 × 10^6^ HT29 cells were injected subcutaneously into mice to induce colon cancer for 7 days. After xenografts reached volumes of 100 mm^3^, AGA extract (200 mg/kg/day) was administered by oral gavage. Tumor size was measured using a caliper and calculated by the formula: volume = length x (width)^2^ /2. After 4 weeks mice were sacrificed with CO_2_ inhalation. Liver and kidney were isolated from mice to evaluate the toxicity of AGA by morphological observations of hematoxylin and eosin-stained.

### Statistical Analysis

The sample sizes of all the data are at least *n* = 3 unless otherwise indicated. The data presented are representative of at least three independent experiments. Statistical analyses were performed using sigma plot software.

## Results

### Anti-proliferative activity of AGA on colon cancer cell

**Fig. 1:**
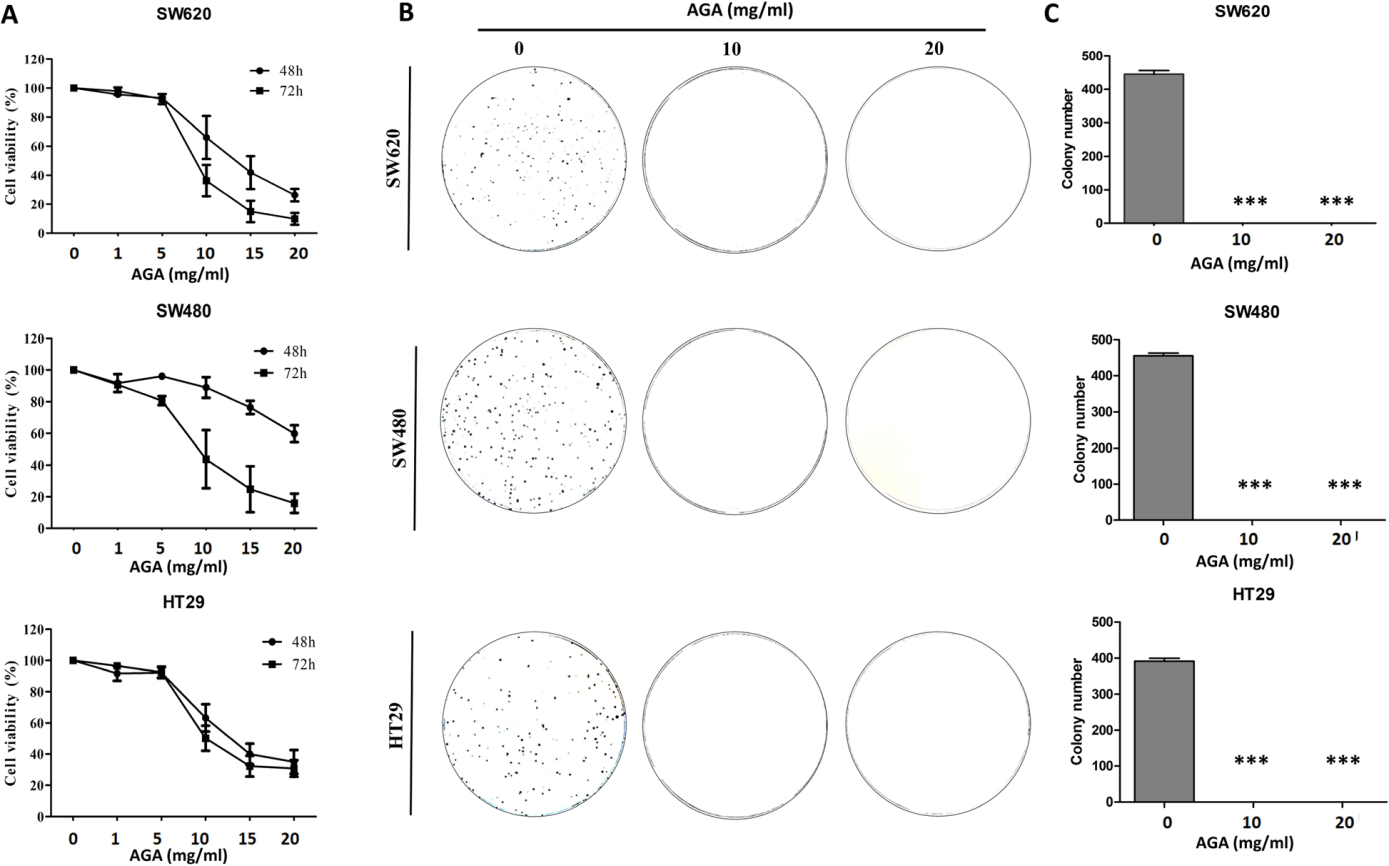
AGA effects on colon cancer cells proliferation and viability. **A **After 24 h of culture, colon cancer cells SW620, SW480, and HT29 were treated with a medium containing vehicle control (0 mg AGA) or increasing concentrations of AGA. Treated and control cells were harvested at 0, 48, and 72 h. Cell proliferation was quantified with MTT Assay. **B **After treating SW620, SW480, and HT29 cells with increasing AGA concentrations (0 to 20 mg) at 37 °C for 72 h, the colony formation was determined along with its quantification (**C**). Results are expressed as the mean percentage of control growth ± SD of three independent experiments with *n* = 3. *p* values were determined by one-way ANOVA. * *p* < 0.05, ** *p* < 0.01, and *** *p* < 0.001

To evaluate the antiproliferative effect of AGA, three different human colon cancer cell lines (SW620, SW480, and HT29), were treated with increasing dosage of AGA for 48 and 72 h. The results from the MTT assay showed that AGA inhibited cell viability of all colon cancer cells in a dose-dependent manner with an IC_50_ value of 10 mg/ml at 72 h (Fig. 1A). Therefore, we chose 0, 10, 20 mg/ml of AGA as experimental dosages for later study. Still, the proliferation of SW620, SW480, and HT29 was evidenced by remarkably reduced colony formation (Fig. 1B) along with its quantification (Fig. 1C).

### AGA inhibits the migration of colon cancer cells

**Fig. 2:**
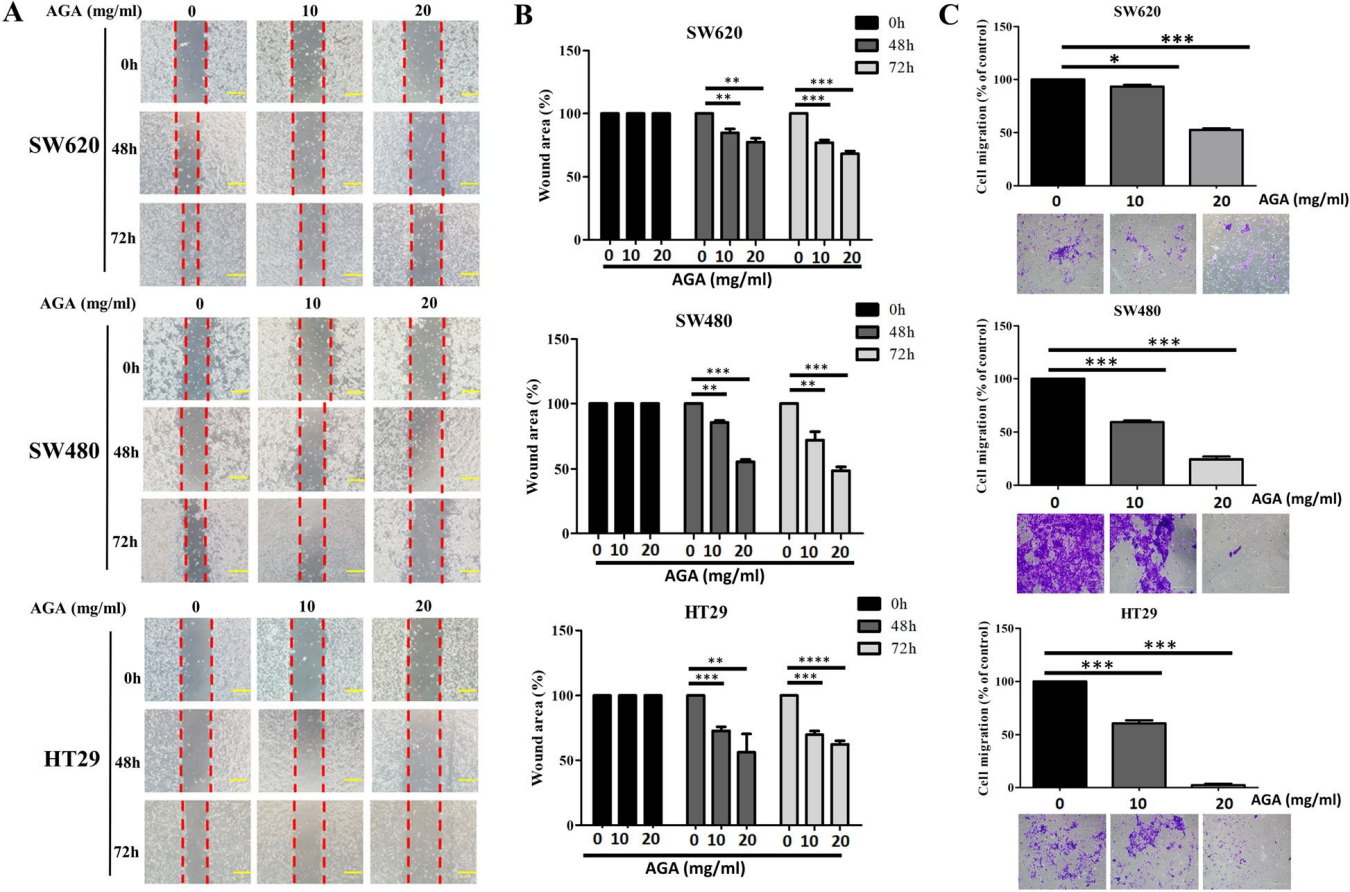
Efficacy of AGA extract on cell migration in colon cancer cells. **A **Cell motility of SW620, SW480, and HT29 upon AGA treatment was determined by wound-healing assay at 0, 10 and 20 mg/mL for 48 and 72 h. Representative photomicrographs (magnification, 100 μm) of wound healing and (**B**) relatively quantified wound area (%). **C **Transwell assay-dependent analysis of the invasive ability of colon cancer cells (magnification, ×100), and their relative qualification. Data are represented in triplicates as mean ± SD

To explore whether AGA extracts exhibit an anti-cancer effect on cell migration, we examined the motility of colon cancer cells via the wound healing assay. Cells with 100% confluence were scratched to create the wounds and were then treated with different dosages of AGA extract (0, 10, and 20 mg/mL); the wound healing was then observed after 48 and 72 h of treatment. Our result showed a significant inhibition of cell motility in AGA-treated SW620, SW480, and HT29 cells in a dose and time dependent manner (Fig. 2A), which has also been confirmed with their relatively quantified wound area (Fig. 2B). Consistent with the wound healing assay, the transwell assay confirmed that the AGA extract treatment resulted in a remarkable decrease in the migration ability of colon cancer cells in a dose-dependent manner, and further validated through their relative quantification (Fig. 2C).

### AGA influence the cell cycle distribution of colon cancer cells

**Fig. 3:**
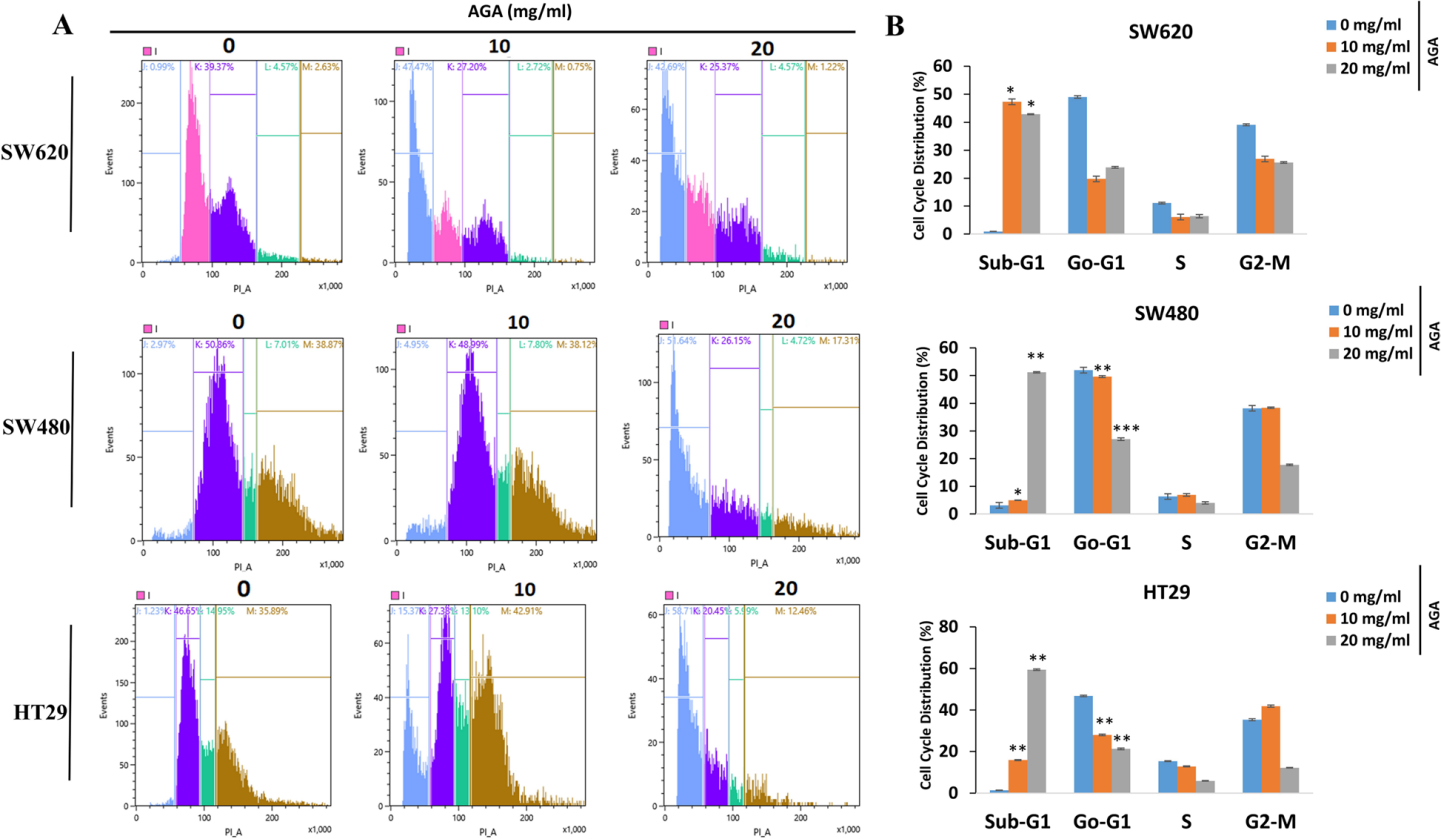
Effects of AGA on cell cycle distribution in colon cancer cell lines. **A** After 24 h of culture, colon cancer cells SW620, SW480, and HT29 were treated with a medium containing vehicle control (0 mg) or increasing concentrations of AGA (10 and 20 mg/ml) for 72 h. Cells were collected and analyzed by flow cytometry. **B** Histograms represent quantitative analysis of cell distribution in the cell cycle phases

Normally, the inhibition of tumor growth is associated with cell cycle modulation. Indeed, previous studies reported that the extracts of the various medicinal plant have the potential to modulate checkpoints at sub-G1 and G2-M of the cell cycle, which contributed to their anti-proliferative activities [22]. To evaluate the effects of AGA on the cell cycle, colon cancer cells were treated with AGA and analyzed by a flow cytometer (Fig. 3A). The quantitative result of each phase in the cell cycle revealed that the sub-G1 phase was significantly increased after AGA treatment in all cell lines, especially at a dose of 20 mg/ml (Fig. 3B).

### AGA extract activates p21-dependent pathway to inhibit CDK proteins in colon cancer cells

**Fig. 4:**
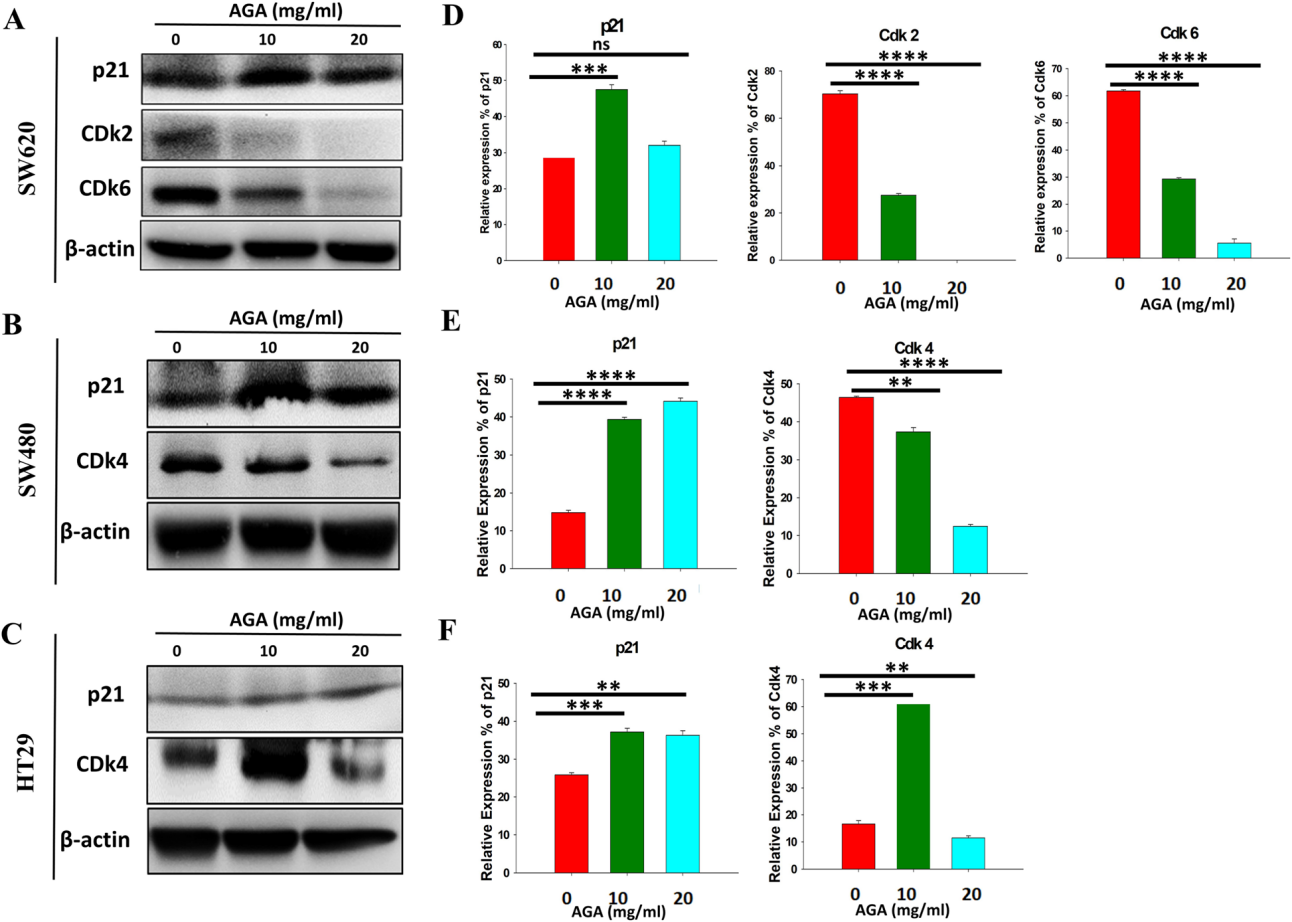
Modulation of key cell cycle regulators with AGA extract in colon cancer cell lines. Immunoblot analysis of p21, cdk2, cdk6, and cdk4 in AGA extract (0 mg, 10 mg, and 20 mg)-treated (**A**) SW620, (**B**) SW480, and (**C**) HT29 cells for 72 h. β-lactin: oading control. **D**-**F** Quantification of western blotting obtained **A**-**C**. Data are expressed as mean fold induction ± SD of three independent experiments. *p* values were determined by one-way ANOVA. * *p* < 0.05, ** *p* < 0.01, and *** *p* < 0.001

Cyclin-dependent kinase (CDK) protein mainly regulates the cell cycle progression through the G1 phase and enters into S-phase. We found that AGA induced cell cycle arrest in the above result by accumulating the sub-G1 phase. To study the molecular mechanism involved in AGA-induced cell cycle arrest, we examine the expression of the p21 protein in preventing the cell cycle. We found that p21 protein expression was increased in SW620, SW480, and HT29 cells after treatment with AGA (Fig. 4). The p21 protein is essential in preventing the cell cycle progression through the G1-S phase transition by forming an inhibitory complex with CDK2, CDK4, and CDK6 [23]. The results showed that AGA treatment decreased the expression of CDK2 and CDK6 in SW620 cells (Fig. 4A), whereas CDK4 in SW480 (Fig. 4B) and HT29 cells (Fig. 4C), respectively. Further, these data were validated through their quantification (Fig. 4D-F) and suggested that AGA induced the cell cycle arrest in colon cancer cells could be through p21-dependent upregulation and inhibited the levels of cell cycle regulators to decrease the kinase activity of CDKs.

### AGA induces apoptotic cell death in colon cancer cells

**Fig. 5:**
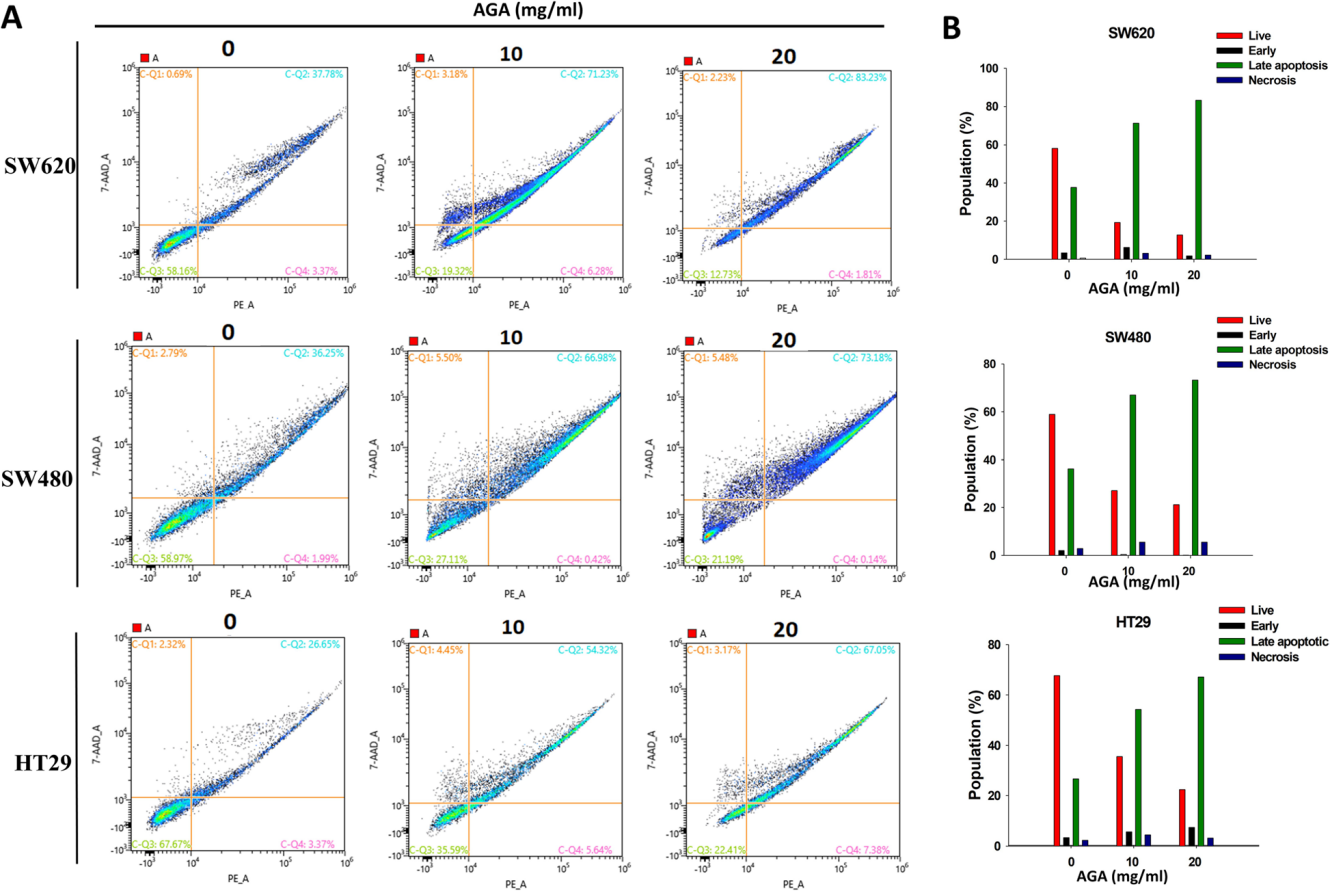
AGA induces apoptosis of colon cancer cells. **A** After 24 h of culture, colon cancer cells SW620, SW480, and HT29 were treated with a medium containing vehicle control (0 mg AGA) or concentrations of AGA (10 to 20 mg/ml) for 72 h and then stained with Annexin V at the end of treatment. **B** The histogram represents the live, early, late, and necrotic phase of apoptosis of colon cancer cells after their treatment with AGA extract and their quantified population

To further confirm whether AGA induces cell death through the apoptosis pathway, colon cancer cells were stained by Annexin/7-ADD to observe the apoptotic population. In our result, a high population of the viable cell was observed in the control group, however, the cell population shifted from viable cells to late apoptosis was observed for all colon cancer cells in a dose-dependent manner (Fig. 5A), which was further confirmed by quantitative results (Fig. 5B).

### AGA extract triggers the pro-apoptotic proteins expression by p53-independent and dependent pathway in colon cancer cells

**Fig. 6:**
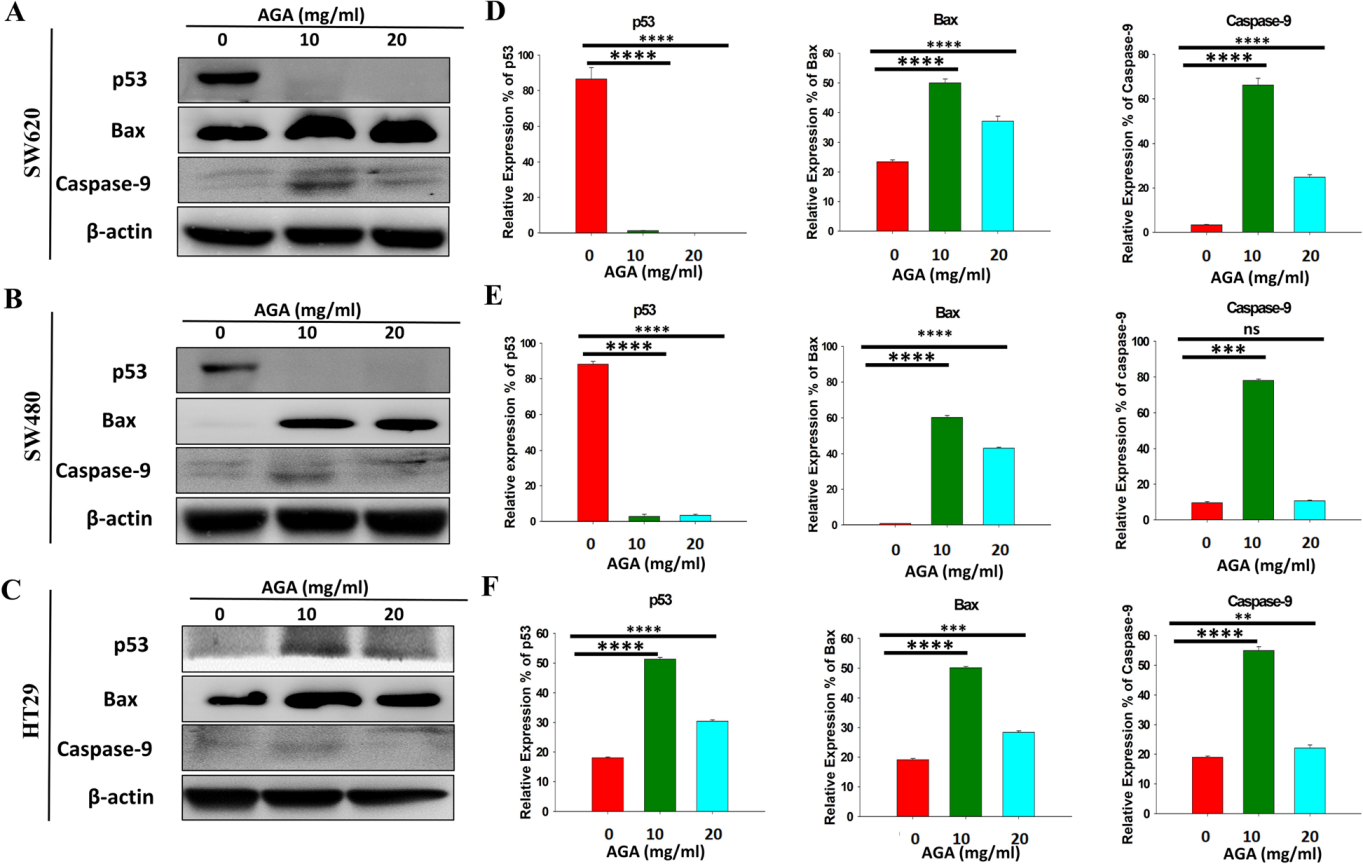
Impact of AGA extract on the expression of apoptosis regulators in colon cancer cells. Immunoblot analysis of p53, Bax, and caspase-9 in AGA extract (0 mg, 10 mg, and 20 mg)-treated (**A**) SW620, **(B)** SW480, and (**C**) HT29 cells for 72 h. β-actin: loading control. **D**-**F** Quantified expression percentage of apoptosis regulator proteins obtained **A**-**C**. Data are expressed as mean fold induction ± SD of three independent experiments. *p* values were determined by one-way ANOVA. * *p* < 0.05, ** *p* < 0.01, and *** *p* < 0.001

To investigate the signaling mechanism of AGA-induced apoptosis in colon cancer cells, we determined the expression of apoptosis-related proteins including p53, Bax, and caspase-9. We found that AGA downregulated the expression of p53 protein in SW620 and SW480 cells (Fig. 6A, B), whereas its expression was upregulated in HT29 cells (Fig. 6C). As apoptosis initiating through a p53-independent or -dependent pathway [22] and further through intrinsic or extrinsic stimulus that activates pro-apoptotic proteins like Bax and caspase-9, our results exhibited that AGA significantly upregulated the expression of Bax and cleaved caspase-9, through a p53-independent pathway in SW620 and SW480, and p53-dependent pathway in HT29, respectively (Fig. 6A-C). These results were further validated by the quantitative expressions of each protein in SW620, SW480, and HT29, respectively (Fig. 6D-F).

### Inhibitory effect of AGA extract on Tumorigenesis in mice

**Fig. 7:**
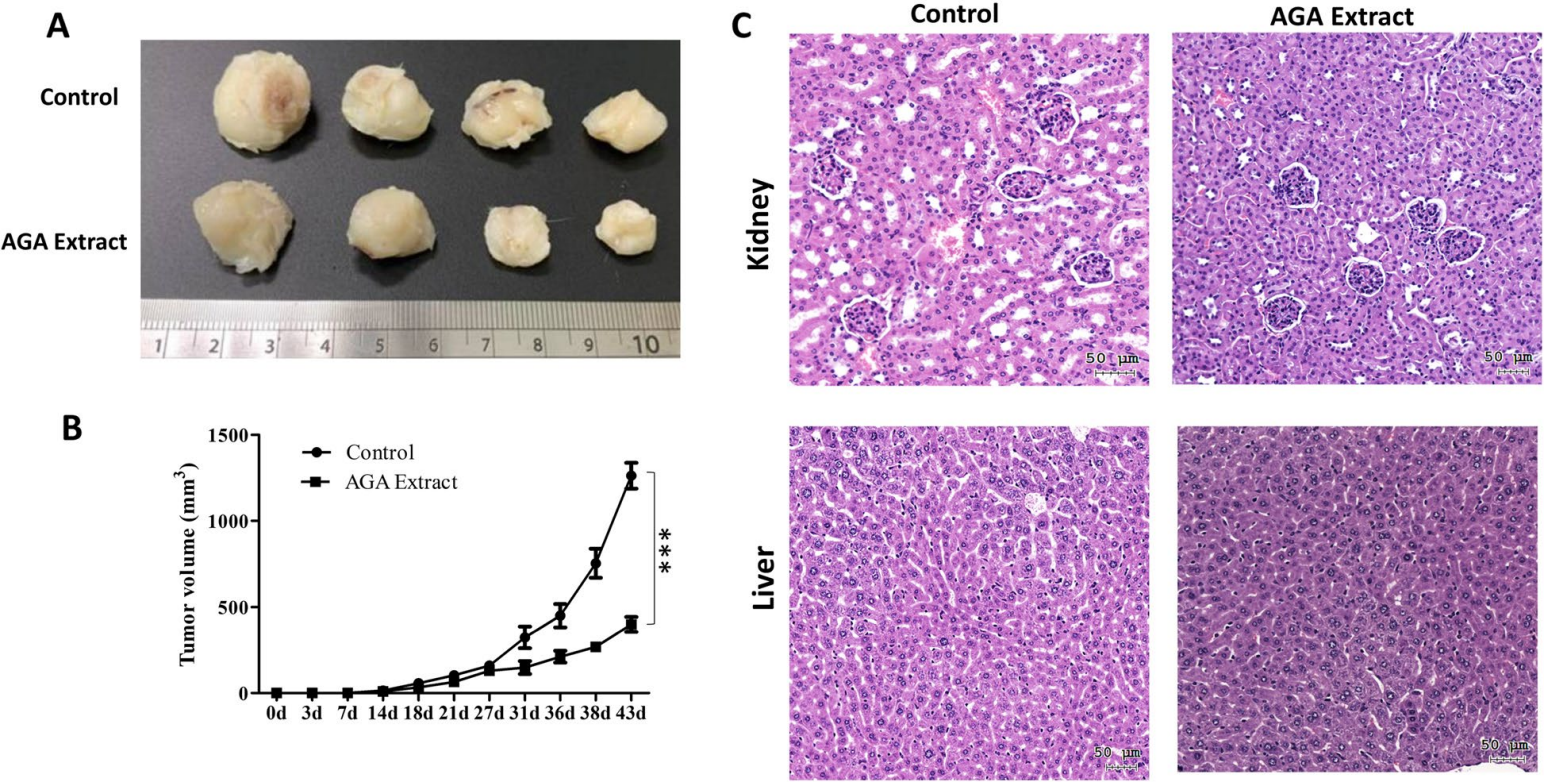
AGA impact on tumorigenesis and organ toxicity. **A** Representative photomicrographs of tumors from HT29 as target cell line and (**B**) its quantified volume derived from mice with colon cancer. **C** AGA- associated renal and hepatic toxicity was assessed through haematoxylin and eosin-stained sections of the kidney and liver. Magnification, 40x (Scale-50 μm)

To assess the in vivo antitumor effects of AGA extract on the initiation and progression of colon cancer, the tumor size and volume were measured in mice after the treatment of AGA extract. We choose HT29 as the target cell line in the animal study. The tumors were excised surgically after the mice were sacrificed after 43 days. We observed reduced tumor size in the AGA-treated group when compared to the control group (Fig. 7A), which was further in line with the significantly suppressed tumor volume (Fig. 7B). We further confirmed the possible adverse events in terms of renal or liver toxicity, through haematoxylin and eosin-stained sections of kidney and liver, respectively. The results showed no morphological changes in the control and AGA-administered groups (Fig. 7C). Taken together, these results demonstrated its in vivo anti-tumor effect, and also excluded the concerns of adverse events caused by AGA therapy.

## Discussion

Apoptosis and cell cycle deregulation is a common features of human cancer. The cancer cells frequently display unscheduled proliferation, migration, and apoptosis [24]. The abnormal expression of many cell cycle and apoptotic proteins drives the biological characteristics of cancer cells. Mainly, kinases are responsible for changing the cell cycle by dysregulating the different checkpoints; their inhibition regulates the cell cycle, which leads to apoptosis and inhibition of cancer cell migration [25]. Therefore, kinases are a potential therapeutic target for cancer cells. However, several data reported that treatment with natural products and chemotherapy in patients with colon cancer had pushed their survival period to almost 20 months, resulting in this kind of treatment being essential for colon cancer treatment [9, 26]. Furthermore, resistance to chemotherapy is a significant challenge to the success of anti-cancer drugs [27]. In colon cancer, dysfunction of p53 expression confers resistance against chemotherapy, and it is a potential target during therapy that critically limit the use of the effective and common herbal drug [15]. To overcome these challenges, our current research mainly focuses on using a combination of three different herbal Chinese medicine: Antler’s, *Ganoderma lucidum*, and *Antrodia camphorata* (AGA). A natural product contains several biomolecules that can change the cell signaling and microenvironment of cancer, thus playing an essential role in combating cancer [28]. Anti-cancerous properties of AGA were reported on oral cancer cell lines, but their effect on colon cancer and related mechanism still need to explore. Here, we demonstrated that AGA could inhibit progression, metastasis, and cell cycle and induce apoptosis in colon cancer. We also reported that p21-dependent and p53-independent/dependent pathways might be involved in inhibiting colon cancer.

In this study, we evaluated the anti-proliferative activity of AGA on SW620, SW480, and HT29 colon cancer cells in a time-dependent manner. The AGA extract showed maximum inhibition in all colon cancer cell lines at 72 h. The previous study also have reported the anti-proliferative activity of AGA against SAS cell lines maximally after 72 h of treatment. Despite the recent advance in the treatment of colon cancer, metastasis remains targeted to inhibit cancer. Cancer cells migrate from the primary site, invade through connective tissue, and penetrate in basement membrane [28]. Even cells migrate through blood vessels and reach distinct organs [29, 30]. Our study showed that AGA extract possessed a potent inhibitory effect on the migration and invasion of SW620, SW480, and HT29 colon cancer cells at 72 h of treatment. These results suggested that AGA could potentially suppress the proliferation and metastasis of colon cancer through inhibition of migration and invasion.

**Fig. 8:**
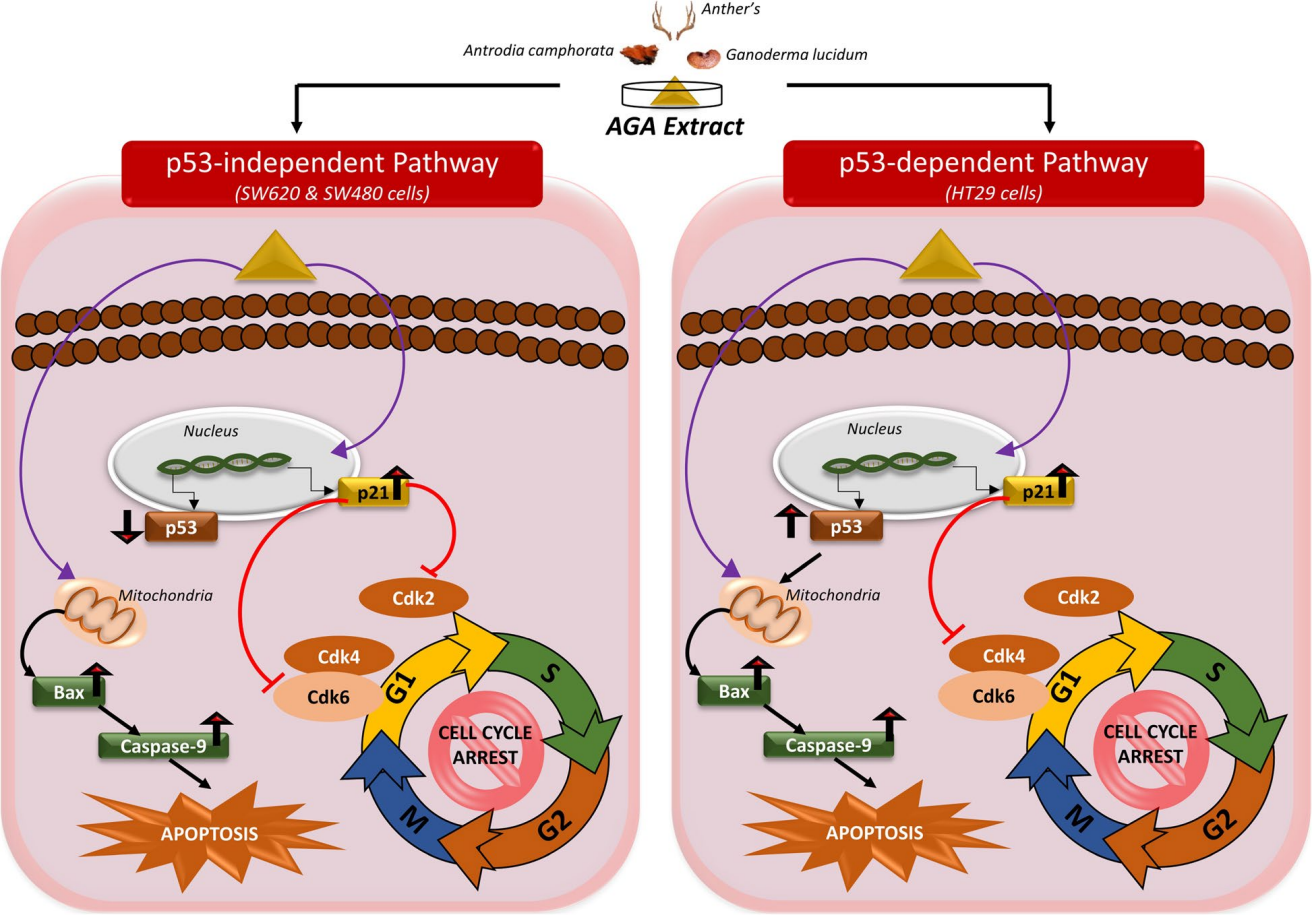
Schematic representation of the possible underlying mechanism of AGA against colon cancer cells

Next, we explored the mechanism of AGA-mediated inhibition of SW620, SW480, and HT29 colon cancer cell growth. Cell cycle regulation and inhibition of apoptosis are essential factors for cancer cell growth [31, 32], and we investigated whether AGA could inhibit cell cycle progression in all colon cancer cells. The result showed an increased sub-G1 phase could be seen after the treatment with AGA extract in SW620, SW480, and HT29 for 72 h. Cyclin-dependent kinase (CDK) protein mainly controlled the cell cycle progression, among which CDK-2, CDK-6, and CDK-4 play an essential role in the G1 to S-phase transition [33]. In cancer cells, for G1 to S-phase transition in the cell cycle, CDK-4 or CDK-6 make a complex with cyclin D, and CDK-2 makes a complex with cyclin-E. The cell cycle CDKs positively and negatively regulated through p53 may also induce the cell cycle arrest at the G1 phase and initiates apoptosis [33, 34]. In response to cancer cell damage, p53 activates the transcription of the p21 protein [35]. After AGA treatment, we found that expression of p21 was increased in all colon cancer cell lines. Moreover, p21 negatively regulates the CDK protein, a universal cell cycle inhibitor that promotes cell cycle arrest in response to various stimulations [36, 37]. Further, we found that expression of CDK2 and CDK6 were significantly decreasing in SW620, and CDK4 expression decreasing in SW480 and HT29, respectively. However, according to Datto et al., TGF-β is capable of directly inducing transcription of p21 proteins; this induction is not dependent on the expression of p53 [38]. Our study has also been in line with the study that showed mimosine could increase the p21 protein level and induce a p53-independent p21 pathway in cancer cells [39]. AGA also inhibits the SAS cells in a p53-independent manner [16]. Therefore, we speculated that AGA inhibits the SW620 and SW480 colon cancer cells through p53-independent, and HT29 colon cancer cell p53-dependent pathways (Fig. 8).

Furthermore, apoptosis is a cell death and defence mechanism, which precisely regulates cell numbers to remove unwanted and potentially hazardous cells. During tumorigenesis, the deregulation of the biochemical pathways controls apoptosis as well as cell proliferation. The p53 protein is essential for the activation of apoptosis in many cancer cells, whereas upregulation of the p53 protein may also inhibit the apoptosis in cancer cells and make them resistant to chemotherapy [40, 41]. In most cancer cells, pro-apoptotic protein induced apoptosis by p53 through intrinsic insults. Even several studies also reported that pro-apoptotic protein also activated without p53 through extrinsic insults that directly induced apoptosis [42]. The Bax protein is localized in the outer mitochondrial membranes and various reports imply that it induced apoptosis by promoting the release of cytochrome c from mitochondria to activate caspase-9 [43]. We found that AGA extract increases the apoptosis in all colon cancer cells by upregulating the expression levels of pro-apoptotic proteins Bax and caspase-9 while downregulating the p53 expression in SW620 and SW480 colon cancer cell line to mediate p53-independent apoptosis. In HT29 colon cancer cells, AGA also increases the expression of pro-apoptotic protein through mediated p53-dependent apoptosis (Fig. 8). A similar observation reported by Wu et al., a specific downregulation of p53 in MCF-7 led to apoptosis [44]. These results are also in line with the study, demonstrating that a herbal extract of *Artocarpus elasticus* and *Paeoniae radix* suppressed cancer cell viability while inducing apoptosis in a dose-dependent manner, protein expression studies revealed significant upregulation of Bax, caspase-9, p21, and downregulation of p53 [45, 46]. These findings suggested that AGA extract mediated inhibition of colon cancer cells induces cell cycle arrest and apoptosis through p53-independent/dependent pathway activation.

To further examine the anti-tumorigenic impact of AGA in colon cancer, we conducted an in vivo study using xenograft nude mice. We observed that AGA could significantly inhibit the growth of xenograft tumors in NOD/SCID mice. In line with our study on *Antrodia camphorata* [16], a previous study has shown that methanol extract from *G. lucidum* could significantly inhibit B16 mouse melanoma growth in vivo [47]. Moreover, we found that AGA herbal extract supplementation showed no effect on the structure and function of the liver and kidneys, as shown by their morphology and the biochemical profile of control and AGA-administered groups, indicating its safety. Collectively, these results demonstrated that AGA extract could be also a better herbal or chemotherapeutic drug for colon cancer. However, it is needed to further evaluate the clinical outcome of this herbal drug in the treatment of colon cancer patients.

## Conclusion

In conclusion, our study demonstrated that AGA extract has potential to inhibit the proliferation, metastasis by inducing apoptosis and G1 phase arrest efficiency by inhibiting the CDK2, CDK6/CDK4 proteins in colon cancer cells. The underlying mechanism of these effects could be mediated through p53-independent/independent pathway (Fig. 8). It is expected that AGA extract a novel herbal anticancer drug in the treatment of colon cancer cells.

## Supplementary Information

**Additional file 1:** **Supplementary figure 1.** Raw images of western blot (including blot images as they are, with the membrane edges and all repeats) represent the AGA (0, 10, and 20 mg) extract effects on three different cell lines SW620, SW480, and HT29 to investigate expression of cell cycle proteins. (A) Represent the cdk4, (B) cdk2, (C) p21, and (D) cdk6 protein expression after AGA extract effect on three colon cancer cell lines. All data were represented with their three repeats; and red colour brackets in original blot images indicates edges of membrane in each images that used in manuscript of figure 4ABC. **Supplementary figure 2. **Raw images of western blot (including blot images as they are, with the membrane edges and all repeats) represent the AGA (0, 10, and 20 mg) extract effects on three different cell lines SW620, SW480, and HT29 to investigate expression of cell cycle proteins. (A) Represent the cdk4, (B) cdk2, (C) p21, and (D) cdk6 protein expression after AGA extract effect on three colon cancer cell lines. All data were represented with their three repeats; and red colour boxes in original blot images indicates that images are used in manuscript of figure 4ABC.** Supplementary figure 3. **Raw images of western blot (including blot images as they are, with the membrane edges and all repeats) represent the AGA (0, 10, and 20 mg) extract effects on three different cell lines SW620, SW480, and HT29 to investigate expression of apoptosis. (A) Represent the p53, (B) Bax, and (C) Caspase 9 protein expression after AGA extract effect on three colon cancer cell lines. All data were represented with their three repeats; and red colour brackets in original blot images indicates edges of membrane in each images that used in manuscript of figure 6ABC. **Supplementary figure 4. **Raw images of western blot (including blot images that used in manuscript) represent the AGA (0, 10, and 20 mg) extract effects on three different cell lines SW620, SW480, and HT29 to investigate expression of apoptosis. (A) Represent the p53, (B) Bax (in bax blot SW480 mention as 20, 10, 0, but in manuscript used as 0, 10, 20), and (C) Caspase 9 protein expression after AGA extract effect on three colon cancer cell lines. All data were represented with their three repeats; and red colour boxes in original blot images indicates that images are used in manuscript of figure 6ABC. **Supplementary figure 5. **Raw images of western blot (including blot images as they are, with the membrane edges and all repeats) represent the AGA (0, 10, and 20 mg) extract effects on three different cell lines SW620, SW480, and HT29 to investigate expression of B-actin (A) Represent the original blot images of B-actin with repeats; red colour brackets in original blot images indicates edges of membrane in each images that used in manuscript of figure 4ABC and figure 6ABC. (B) Original blot images of B-actin with repeats, red colour boxes in original blot images indicates that images are used in manuscript of 4ABC and figure 6ABC.

## Data Availability

All data analysed or generated during the study are included in this published article.
