# Supplementary material for: AGA induces sub-G1 cell cycle arrest and apoptosis in human colon cancer cells through p53-independent/p53-dependent pathway
Source: BMC Cancer. 2023 Jan 2;23:1. doi: 10.1186/s12885-022-10466-x (PMC9808967; doi:10.1186/s12885-022-10466-x)
Supplement: Supplementary file 1 — Additional file 1: Supplementary figure 1. Raw images of western blot (including blot images as they are, with the membrane edges and all repeats) represent the AGA (0, 10, and 20 mg) extract effects on three different cell lines SW620, SW480, and HT29 to investigate expression of cell cycle proteins. (A) Represent the cdk4, (B) cdk2, (C) p21, and (D) cdk6 protein expression after AGA extract effect on three colon cancer cell lines. All data were represented with their three repeats; and red colour brackets in original blot images indicates edges of membrane in each images that used in manuscript of figure 4ABC. Supplementary figure 2. Raw images of western blot (including blot images as they are, with the membrane edges and all repeats) represent the AGA (0, 10, and 20 mg) extract effects on three different cell lines SW620, SW480, and HT29 to investigate expression of cell cycle proteins. (A) Represent the cdk4, (B) cdk2, (C) p21, and (D) cdk6 protein expression after AGA extract effect on three colon cancer cell lines. All data were represented with their three repeats; and red colour boxes in original blot images indicates that images are used in manuscript of figure 4ABC. Supplementary figure 3. Raw images of western blot (including blot images as they are, with the membrane edges and all repeats) represent the AGA (0, 10, and 20 mg) extract effects on three different cell lines SW620, SW480, and HT29 to investigate expression of apoptosis. (A) Represent the p53, (B) Bax, and (C) Caspase 9 protein expression after AGA extract effect on three colon cancer cell lines. All data were represented with their three repeats; and red colour brackets in original blot images indicates edges of membrane in each images that used in manuscript of figure 6ABC. Supplementary figure 4. Raw images of western blot (including blot images that used in manuscript) represent the AGA (0, 10, and 20 mg) extract effects on three different cell lines SW620, SW480, and HT29 to investigate [file 12885_2022_10466_MOESM1_ESM.docx]

**Supplementary Data**

**Raw/Original Blot images of each western blot data mention in our manuscript**


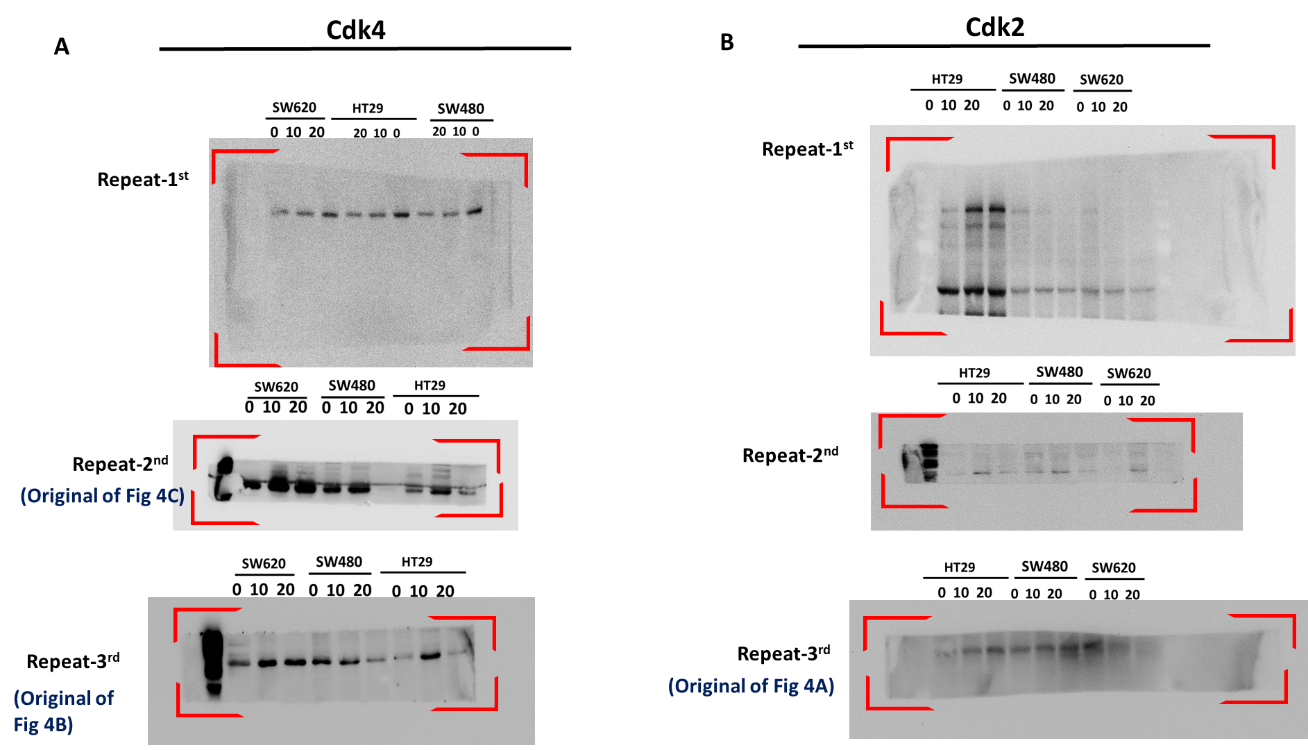


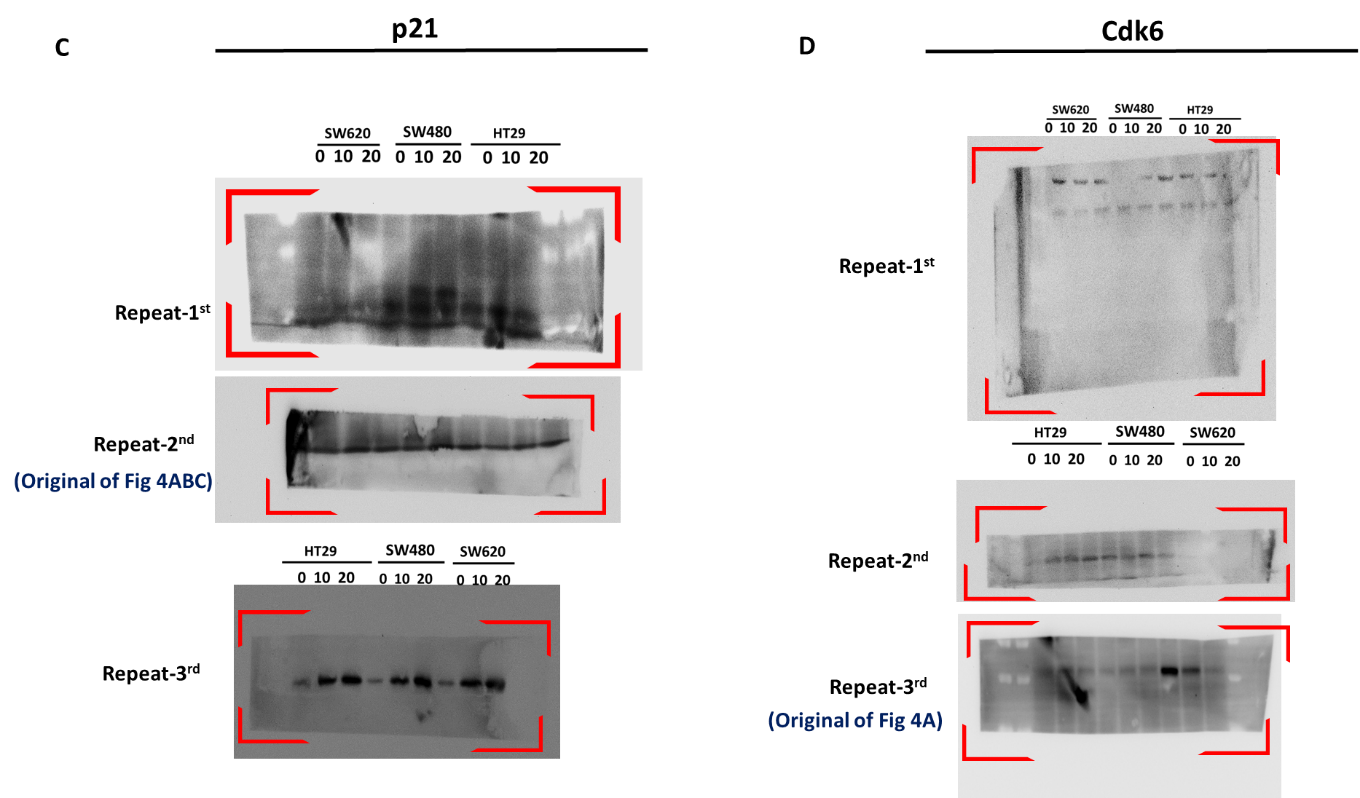


**Supplementary figure 1:** Raw images of western blot **(including blot images as they are, with the membrane edges and all repeats)** represent the AGA (0, 10, and 20 mg) extract effects on three different cell lines SW620, SW480, and HT29 to investigate expression of cell cycle proteins. (A) Represent the cdk4, (B) cdk2, (C) p21, and (D) cdk6 protein expression after AGA extract effect on three colon cancer cell lines. All data were represented with their three repeats; and **red colour brackets in original blot images indicates edges of membrane in each images that used in manuscript of figure 4ABC.**


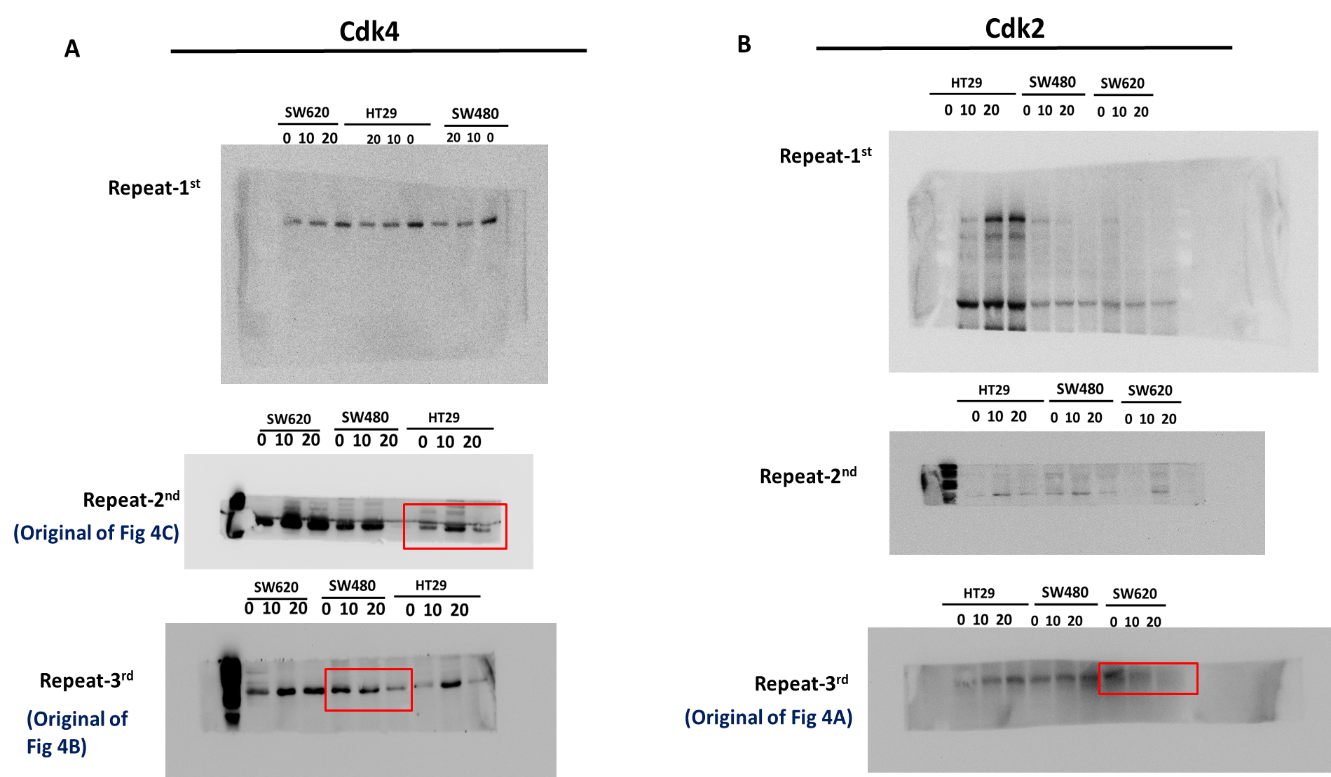


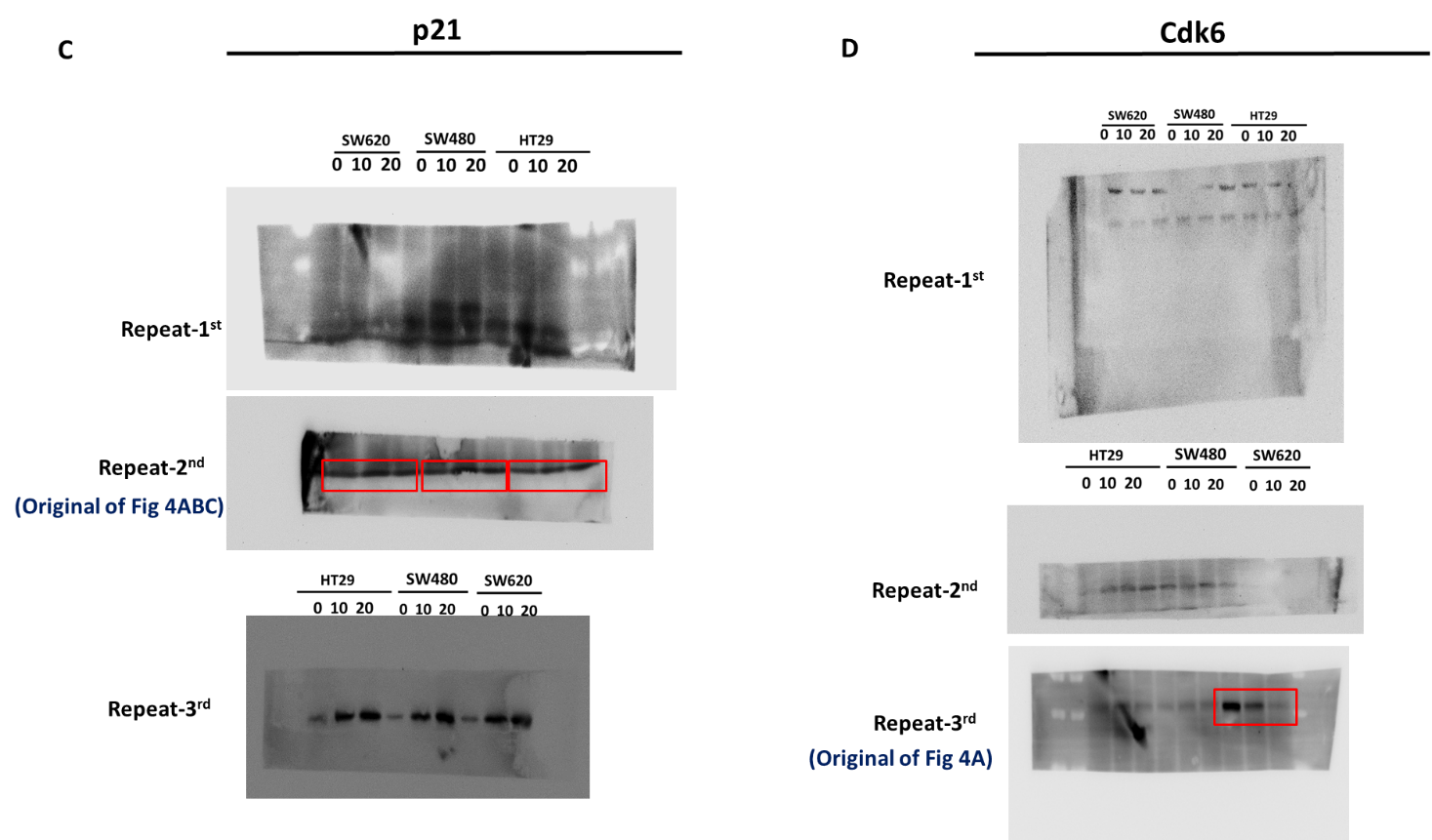


**Supplementary figure 2:** Raw images of western blot **(including blot images as they are, with the membrane edges and all repeats)** represent the AGA (0, 10, and 20 mg) extract effects on three different cell lines SW620, SW480, and HT29 to investigate expression of cell cycle proteins. (A) Represent the cdk4, (B) cdk2, (C) p21, and (D) cdk6 protein expression after AGA extract effect on three colon cancer cell lines. All data were represented with their three repeats; and **red colour boxes in original blot images indicates that images are used in manuscript of figure 4ABC.**


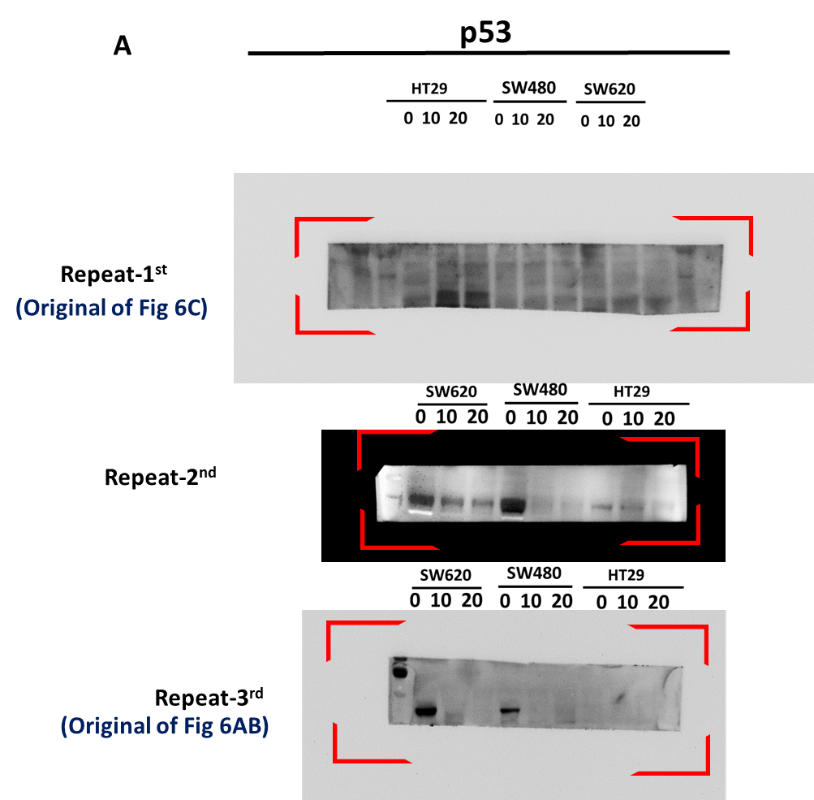


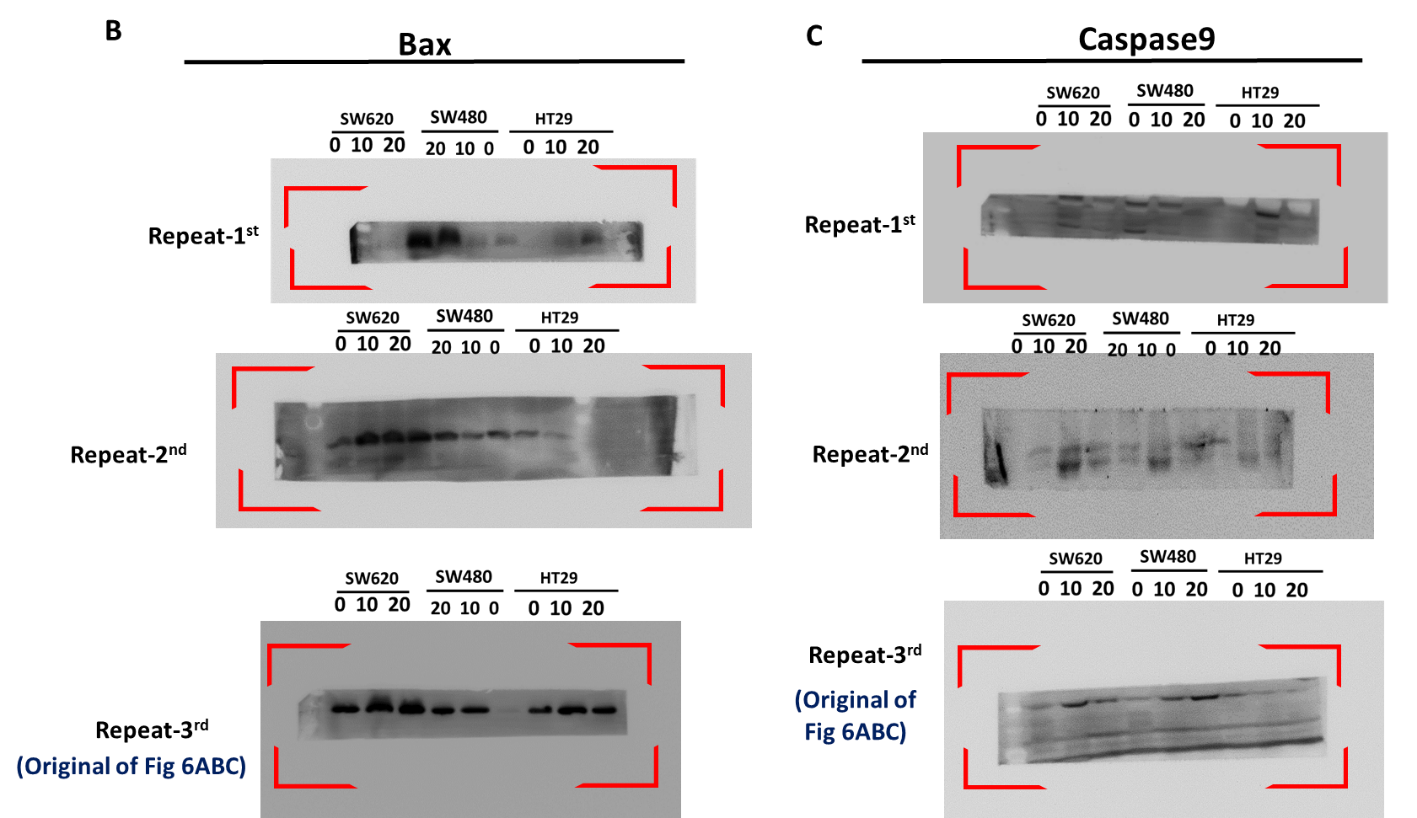


**Supplementary figure 3:** Raw images of western blot (including blot images as they are, with the membrane edges and all repeats) represent the AGA (0, 10, and 20 mg) extract effects on three different cell lines SW620, SW480, and HT29 to investigate expression of apoptosis. (A) Represent the p53, (B) Bax, and (C) Caspase 9 protein expression after AGA extract effect on three colon cancer cell lines. All data were represented with their three repeats; and **red colour brackets in original blot images indicates edges of membrane in each images that used in manuscript of figure 6ABC.**


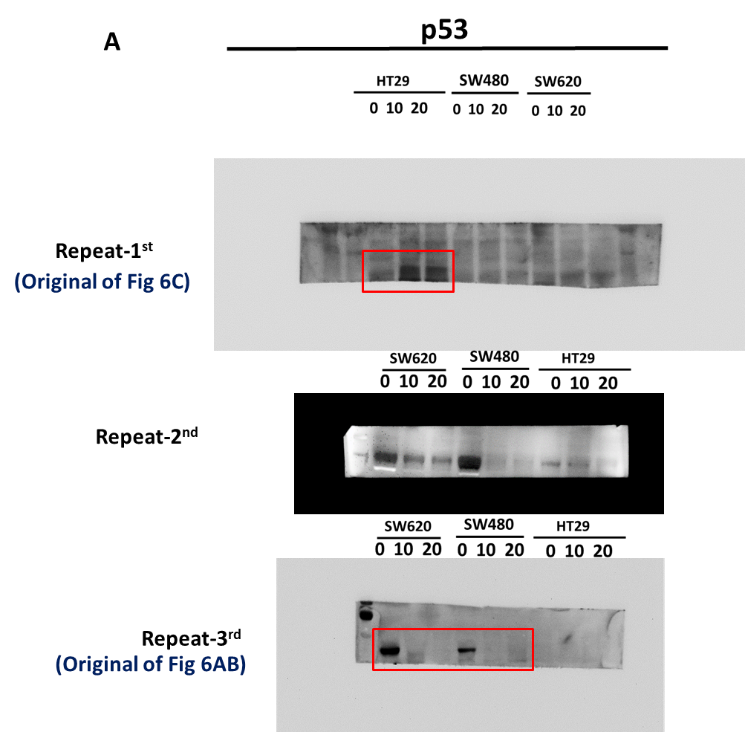


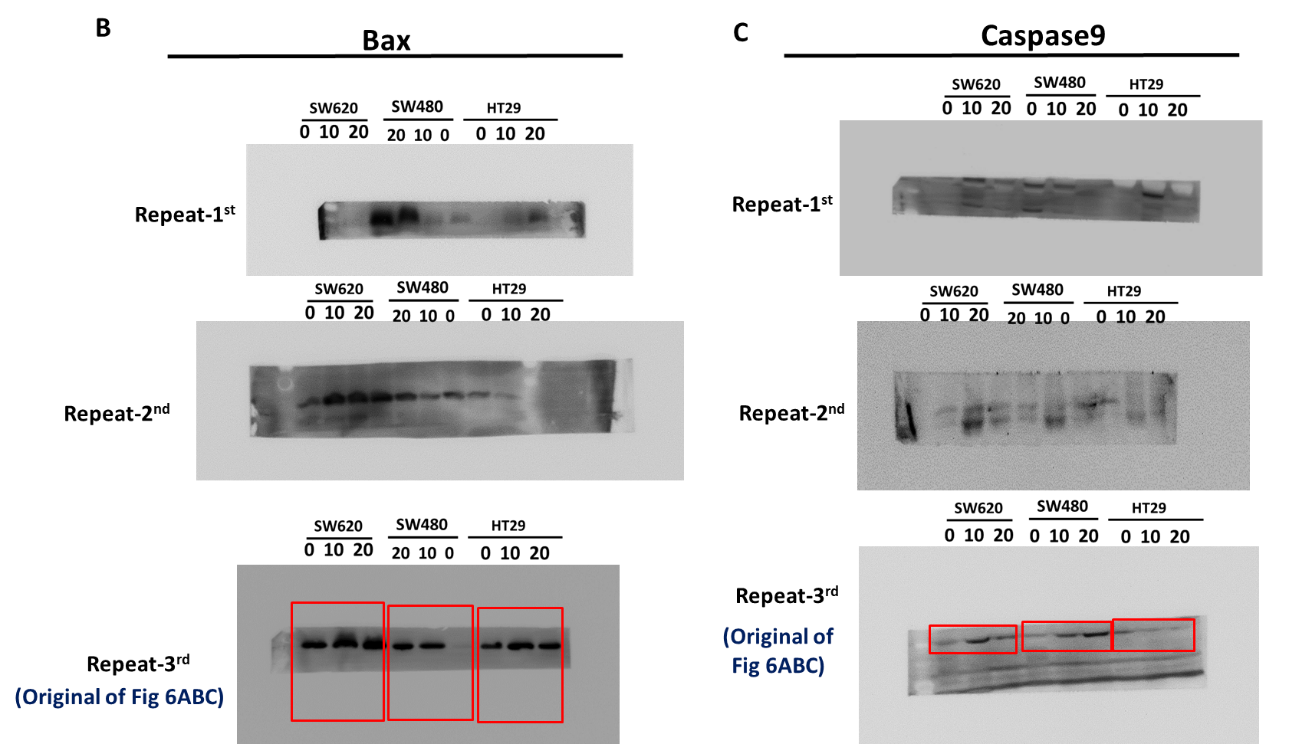


**Supplementary figure 4:** Raw images of western blot (including blot images that used in manuscript) represent the AGA (0, 10, and 20 mg) extract effects on three different cell lines SW620, SW480, and HT29 to investigate expression of apoptosis. (A) Represent the p53, (B) Bax **(in bax blot SW480 mention as 20, 10, 0, but in manuscript used as 0, 10, 20)**, and (C) Caspase 9 protein expression after AGA extract effect on three colon cancer cell lines. All data were represented with their three repeats; **and red colour boxes in original blot images indicates that images are used in manuscript of figure 6ABC.**


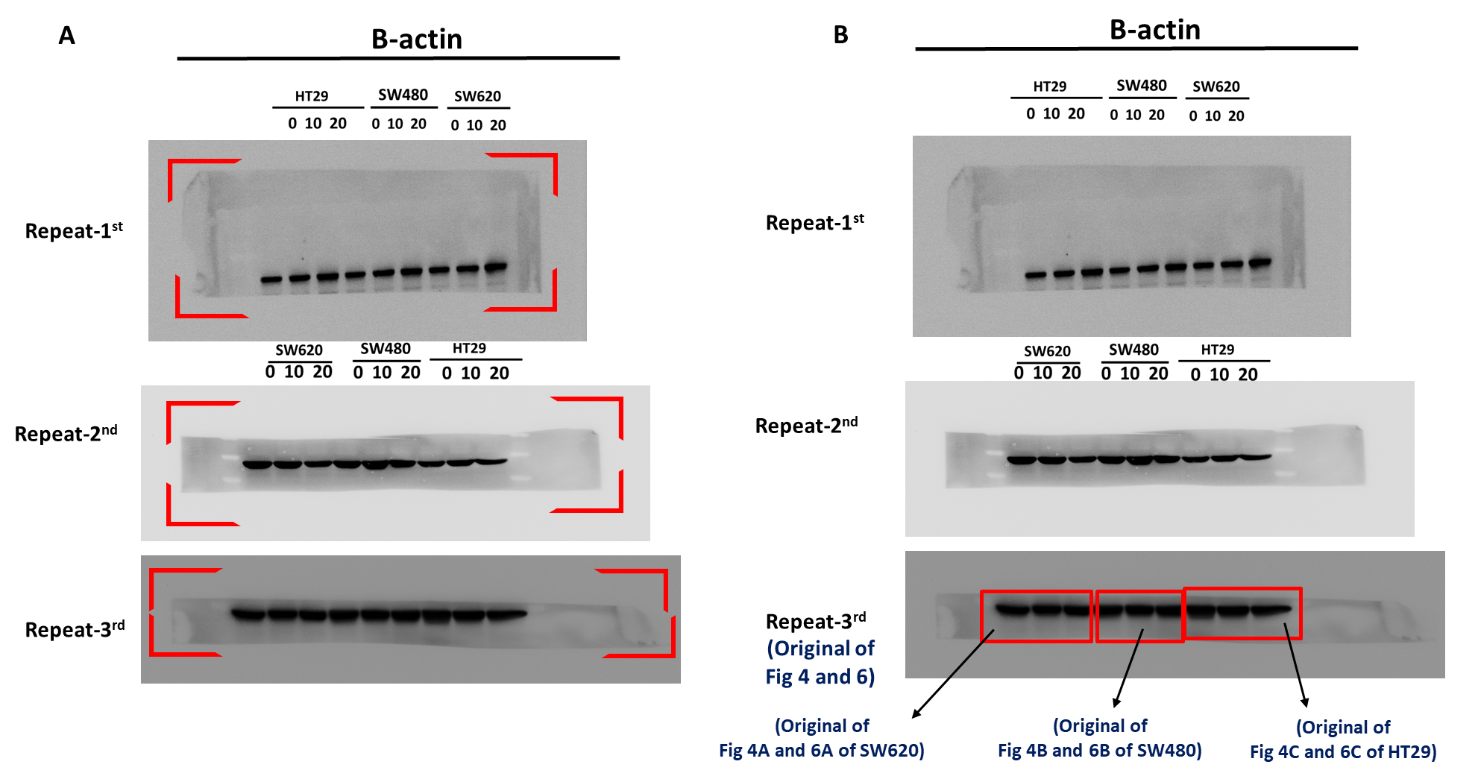


**Supplementary figure5:**  Raw images of western blot (including blot images as they are, with the membrane edges and all repeats) represent the AGA (0, 10, and 20 mg) extract effects on three different cell lines SW620, SW480, and HT29 to investigate expression of B-actin (A) Represent the original blot images of B-actin with repeats; **red colour brackets in original blot images indicates edges of membrane in each images that used in manuscript of figure 4ABC and figure 6ABC.** (B) Original blot images of B-actin with repeats, **red colour boxes in original blot images indicates that images are used in manuscript of 4ABC and figure 6ABC.**
